# Accuracy and Precision of Tidal Wetland Soil Carbon Mapping in the Conterminous United States

**DOI:** 10.1038/s41598-018-26948-7

**Published:** 2018-06-21

**Authors:** James R. Holmquist, Lisamarie Windham-Myers, Norman Bliss, Stephen Crooks, James T. Morris, J. Patrick Megonigal, Tiffany Troxler, Donald Weller, John Callaway, Judith Drexler, Matthew C. Ferner, Meagan E. Gonneea, Kevin D. Kroeger, Lisa Schile-Beers, Isa Woo, Kevin Buffington, Joshua Breithaupt, Brandon M. Boyd, Lauren N. Brown, Nicole Dix, Lyndie Hice, Benjamin P. Horton, Glen M. MacDonald, Ryan P. Moyer, William Reay, Timothy Shaw, Erik Smith, Joseph M. Smoak, Christopher Sommerfield, Karen Thorne, David Velinsky, Elizabeth Watson, Kristin Wilson Grimes, Mark Woodrey

**Affiliations:** 1 Smithsonian Environmental Research Center, Edgewater, USA; 2 USGS, National Research Program, Water Resources Division, Menlo Park, USA; 3 USGS, Volunteer, Sioux Falls, USA; 4 Silvestrum Climate Associates, LLC, Mill Valley, USA; 5 University of South Carolina, Columbia, USA; 6 Florida International University, Miami, USA; 7 University of San Francisco, San Francisco, USA; 8 USGS, California Water Science Center, Sacramento, USA; 9 San Francisco State University and San Francisco Bay National Estuarine Research Reserve, San Francisco, USA; 10 USGS, Woods Hole Coastal and Marine Science Center, Woods Hole, USA; 11 USGS, Western Ecological Research Center, Vallejo, USA; 12 University of South Florida, Tampa, USA; 13 Coastal and Hydraulics Laboratory, U.S. Army Engineer Research and Development Center, Vicksburg, USA; 14 University of California, Los Angeles, USA; 15 Guana Tolomato Matanzas National Estuarine Research Reserve, Ponte Vedra Beach, USA; 16 Delaware National Estuarine Research Reserve, Dover, USA; 17 Asian School of the Environment, Nanyang Technical University, Singapore, Singapore; 18 Earth Observatory of Singapore and Nanyang University, Singapore, Singapore; 19 Florida Fish & Wildlife Conservation Commission, Fish & Wildlife Research Institute, St. Petersburg, USA; 20 Virginia Institute for Marine Sciences, Gloucester Point, USA; 21 North Inlet-Winyah Bay National Estuarine Research Reserve, Georgetown, USA; 22 University of Delaware, School of Marine Science and Policy, Newark, USA; 23 Department of Biodiversity, Earth & Environmental Sciences and The Academy of Natural Sciences, Drexel University, Philadelphia, USA; 24 University of the Virgin Islands and Wells National Estuarine Research Reserve, St. Thomas, USA; 25 Grand Bay National Estuarine Research Reserve and Mississippi State University, Moss Point, USA

## Abstract

Tidal wetlands produce long-term soil organic carbon (C) stocks. Thus for carbon accounting purposes, we need accurate and precise information on the magnitude and spatial distribution of those stocks. We assembled and analyzed an unprecedented soil core dataset, and tested three strategies for mapping carbon stocks: applying the average value from the synthesis to mapped tidal wetlands, applying models fit using empirical data and applied using soil, vegetation and salinity maps, and relying on independently generated soil carbon maps. Soil carbon stocks were far lower on average and varied less spatially and with depth than stocks calculated from available soils maps. Further, variation in carbon density was not well-predicted based on climate, salinity, vegetation, or soil classes. Instead, the assembled dataset showed that carbon density across the conterminous united states (CONUS) was normally distributed, with a predictable range of observations. We identified the simplest strategy, applying mean carbon density (27.0 kg C m^−3^), as the best performing strategy, and conservatively estimated that the top meter of CONUS tidal wetland soil contains 0.72 petagrams C. This strategy could provide standardization in CONUS tidal carbon accounting until such a time as modeling and mapping advancements can quantitatively improve accuracy and precision.

## Introduction

Tidal wetlands, herein including saltmarshes, tidal freshwater wetlands, and tidally influenced forests such as mangroves, are a substantial global sink of organic carbon (C)^1–4^. Mapping tidal carbon stocks and fluxes is challenging, with substantial implications for ecology^5^, carbon markets^6,7^, resiliency^8,9^, and greenhouse gas (GHG) inventorying^10^.

Tidal wetlands store carbon in their soil organic matter when they are stable and release carbon when they are degrading. Organic matter produced *in-situ* is deposited primarily by root addition into shallow anoxic soils^11^. As sea level rises, organic deposition, as well as inorganic sediment deposition, contribute new soil mass that, under the right conditions, allow the wetland surface to vertically accrete and gain elevation in equilibrium with relative sea-level rise^12,13^. Long-term storage properties are variable and depend on salinity, flooding, plant type, and microbial community activity^14^. Tidal wetlands can be a major source of carbon emissions when the soil is lost to erosion^15^, or other disturbances^16,17^. Processes such as drainage or diking can result in direct oxidation of soil carbon or the emission of methane depending on soil type, inundation, and salinity^18^. Erosion results in export of particulate and dissolved organic carbon to other aquatic systems, a portion of which is oxidized and returned to the atmosphere^19^.

In order to both evaluate existing carbon stocks at sub-national to local scales, and estimate emissions from tidal wetlands that are lost during erosion and degradation events, we require accurate and precise soil carbon mapping strategies. We refer to accuracy throughout as the average difference between mapped and reference values. The lack of accuracy is referred to throughout as bias, also commonly referred to as systematic error^20^. We refer to precision throughout as the agreement among repeated comparisons of mapped and reference values. The lack of precision is referred to throughout as imprecision, also known as random error^20^.

Herein we discuss three types of strategies for estimating carbon stocks: applying average carbon stock values from syntheses of soil core data, applying models fit using empirical data and applied spatially using soil, vegetation and salinity maps, and relying on independently generated soil carbon maps that intersect with mapped tidal wetlands. The International Panel on Climate Change (IPCC)’s 2013 *Wetlands Supplement*^10^ to the 2006 assessment report provides global default values based on a literature review. It also provides guidance for ‘higher tier’ analyses utilizing country-specific data, such as a more extensive and thorough review of country-specific soil core data^21,22^ or the use of soils maps^23^. The IPCC *Wetland Supplement* guidance recommends disaggregating wetland emissions based on soil type (organic- and mineral-dominated), as well as by vegetation community, salinity, and climate type. However, the relative importance of these factors and the efficacy of applying separate estimates have not been evaluated at the scale of the conterminous U.S. (CONUS).

Morris *et al*.^24^ recently presented an ‘ideal mixing model’, which describes the physical and volume-limited nature of bulk density in tidal wetland soils. Bulk density and organic matter content are not independent variables; instead, bulk density is a predictable function of organic matter^25,26^, the product of the ‘self-packing densities’ of organic and mineral soil fractions^24^. Although Morris *et al*.^24^ fit this model to describe constraints on tidal wetlands’ resilience to relative sea-level rise, it also has important ramifications for carbon monitoring as organic matter self-packing density defines an effective upper-limit for likely ranges of observable organic matter density.

Hinson *et al*.^23^ utilized the United States Department of Agriculture (USDA)’s Soil Survey Geographic Database (SSURGO) for a CONUS-wide stock assessment, independent of the previously described soil core syntheses. SSURGO is a CONUS-wide series of high resolution soil maps^27^ that link soil classifications and descriptions to tables populated with associated bulk density and % organic matter^28^ depth series information. However, the underlying information used to populate SSURGO soils maps with organic matter content and bulk density values are not necessarily empirical. They can be based on laboratory measurements, or can be assembled from literature, interviews with experts, or interpreted using a soil scientist’s expert judgement^27^. Hinson *et al*.^23^ were not able to perform a full accuracy assessment of their maps because data were not readily available through the literature. One study provided a regional independent validation for SSURGO carbon data for tidal wetlands, among other land cover types, in Louisiana^29^. The study observed a weak positive correlation in organic matter content between SSURGO and independently collected soil cores but did not assess the accuracy of SSURGO-based carbon stock maps^29^.

In the absence of, and with an interest in developing, a robust national-scale strategy for estimating tidal wetland carbon stocks, our goals are twofold. First, we evaluated the efficacy of IPCC *Wetlands Supplement* guidance for reporting and applying soil carbon stock values based on soil type, climate type, and salinity and vegetation. Second we evaluated whether or not more complex, spatially-explicit approaches improved precision and accuracy over a simpler strategy, applying a single mean value based on an extensive empirical dataset.

## Results

### Soil Core Dataset Description

**Figure 1:**
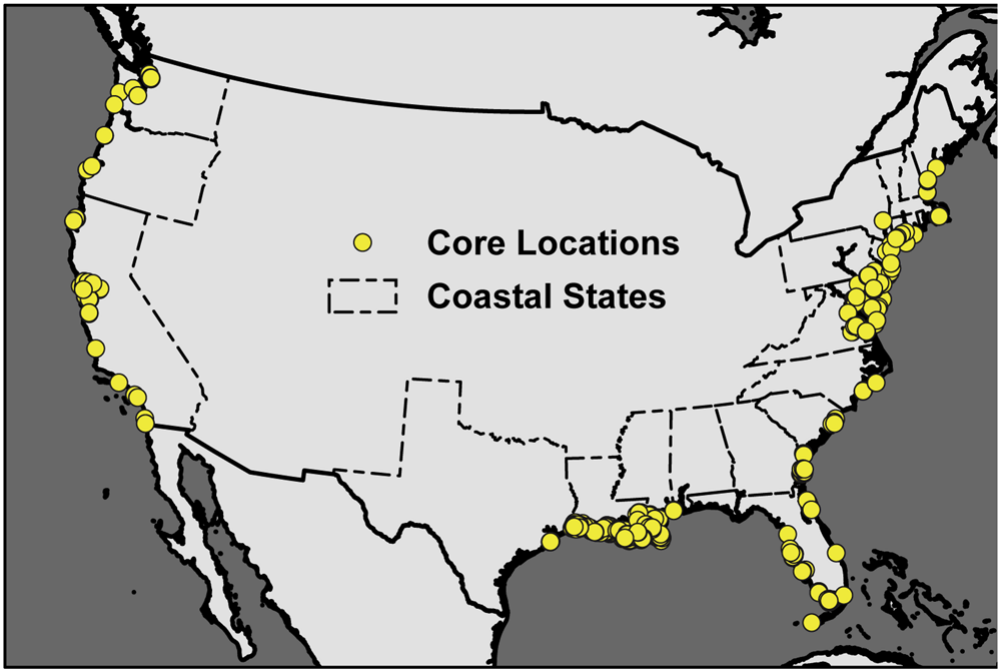
Conterminous United States map showing the locations of soil cores making up the empirical datasets used in this paper. Coastal state borders are modified from public domain 2014-census based 300 m resolution state shapefiles (http://gis.ucla.edu/geodata/dataset/states). Continent borders are modified from ESRI World Continents shapefiles version 10.3 (http://gis.ucla.edu/geodata/dataset/continent_ln).

We assembled a spatially explicit database totaling 1959 soil cores from 49 different studies across CONUS (Fig. 1; Supplemental Table 1). The dataset was dominated by estuarine emergent wetlands (n = 1533), but also contained tidal palustrine emergent (n = 157), estuarine forested and scrub/shrub (n = 46), and tidal palustrine forested and scrub/shrub (n = 87). 134 cores did not have enough accompanying meta-data for us to make this distinction. The empirical dataset was spatially representative with 18 of the 22 coastal CONUS states included.

**Figure 2:**
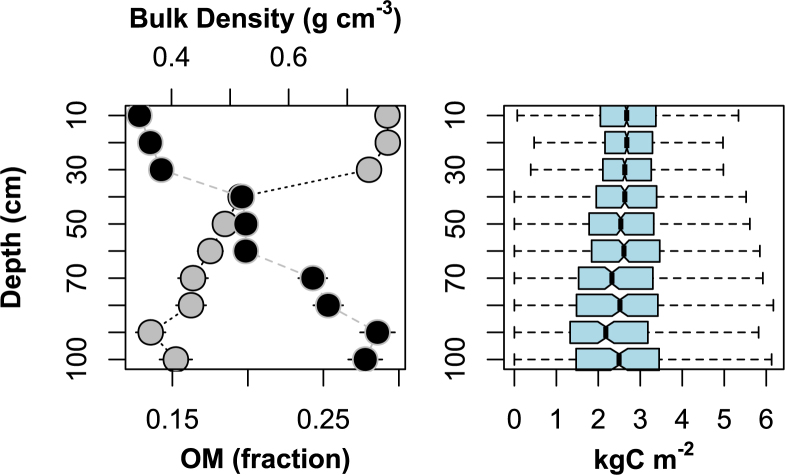
Depth profiles of organic matter (OM; gray circles), bulk density (black circles), and carbon (C) mass (bars). Lines indicate standard error of the mean for bulk density, OM. For carbon mass, the central bars represent the median, box edges the 1st and 3rd quartiles, and whiskers the remaining data distribution excluding outliers, defined herein as 1.5 times the interquartile range.

Average bulk density increased with depth, ranging from 0.35 ± 0.01 to 0.73 ± 0.03 s.e. g cm^−3^, and average organic matter content decreased with depth and ranging from 29 ± 0.5 to 15 ± 1.1 s.e. % (Fig. 2). The two patterns offset each other such that average carbon mass varied little with depth (Fig. 2). Standard error of mean (s.e.) organic matter content, bulk density, and carbon mass all increased with depth as the number of observations decreased from 1959 for the 0 to 10 cm increment to 231 for 90 to 100 cm.

**Figure 3:**
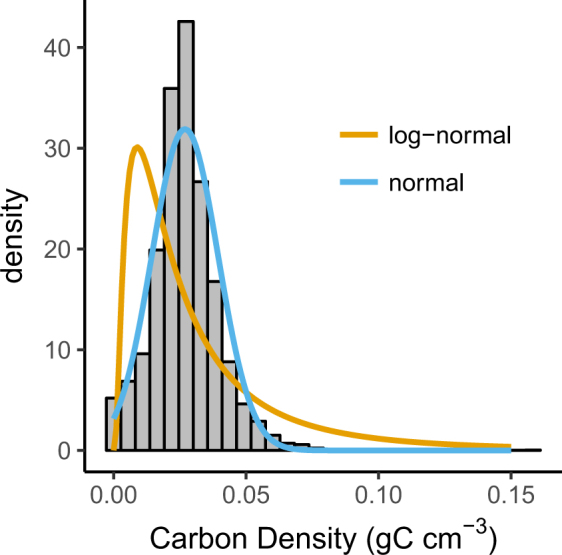
Total probability density distribution of all depth increments for all cores. Although the Intergovernmental Panel on Climate Changes’ Wetlands Supplement reports means and confidence interval data of log-transformed data, our data clearly follows a truncated normal distribution.

We described carbon density using mean and standard deviation (s.d.) assuming a truncated normal distribution in which values could not be lower than 0. The fit of this distribution was an improvement over a log-normal distribution (Fig. 3). For our assembled reference data, average carbon density over 1 m depth^10^ was 0.027 gC cm^−3^ (s.d. = 0.013, n = 8280 samples, s.e. = 1.4 E-4).

### Model Fitting

Model fitting occurred in three major steps: fitting the ideal mixing and organic matter density models, determining an appropriate threshold for categorizing organic- and mineral- dominated soils, and fitting two mixed effects models one with soil type as an independent variable (model 1) and one without (model 2), to describe the major categorical trends and effect sizes within the data.

**Figure 4:**
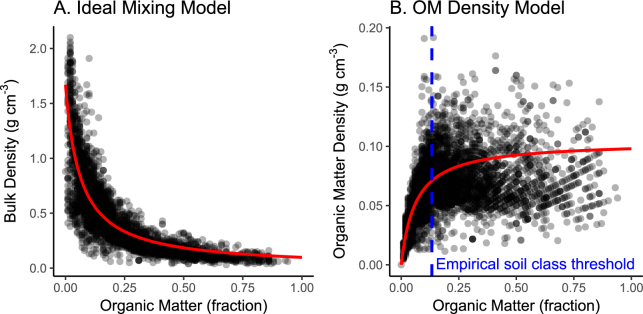
The ideal mixing model and a modification of the ideal mixing model estimating organic matter density as a function of fraction organic matter. Solid red lines represent modeled values. Blue dashed line represents an empirical threshold separating organic and mineral-dominated soil classes determined by a segmented regression.

For the ideal mixing model and organic matter density models (Fig. 4), the self-packing density of organic matter (k1) was 0.098 g cm^−3^ ± s.e. 0.001 (p < 0.0001) and inorganic self-packing density (k2) was 1.67 g cm^−3^ ± s.e. 0.025 (p < 0.0001). When the mixing model was fit to separate 10 cm depth intervals, k1 ranged between 0.086 to 0.146 g cm^−3^ and k2 ranged between 1.34 to 2.32 g cm^−3^; neither k1 nor k2 exhibited a trend with depth to 1 m.

The organic matter density model is useful for describing variability along a spectrum of soils types. However soils are typically mapped using the binary categories of organic- and mineral-dominated. We detected a significant threshold at 13.2% organic matter (95% CI: 12.3% to 13.6%; Fig. 4). Applying this threshold described more variance (R^2^ = 0.30, p < 0.0001) in the carbon mass data than the prescribed definition of either >20% (R^2^ = 0.22, p < 0.0001) or >35% (R^2^ = 0.10, p < 0.0001).

In what we refer to throughout as ‘model 1’, submitter had a random effect of 6.24 E-4 gC cm^−3^. When normalized to the s.d. of the calibration dataset (σ_c_) this equals 0.48 σ_c_ (Table 1). In model 1, soil type, climate type, salinity and vegetation type, and some interactive effects had the highest explanatory power relative to parsimony over all other possible permutations of the model (Table 1). Depth interval was not a significant factor in model 1.

**Table 1:** Structure, submitter random effect size, Akaike’s Information Criterion for small sample size (AICc), and Pseudo R^2^ values for Model 1, which considered all mappable independent variables (soil type, climate, salinity and vegetation type (salVeg), and depth), and Model 2, which excluded soil type.

Model	Structure	n	Standardized Random Effect (σ_c_)	Pseudo R^2^	AICc
Model 1	carbon density~climate + salVeg + soil + (1 | submitter) + climate:salVeg + climate:soil + salVeg:soil + climate:salVeg:soil	3536	0.49	0.51	7278
Model 2	carbon density~climate + depth + salVeg + (1 | submitter) + climate:salVeg	3536	1.04	0.32	8454

**Figure 5:**
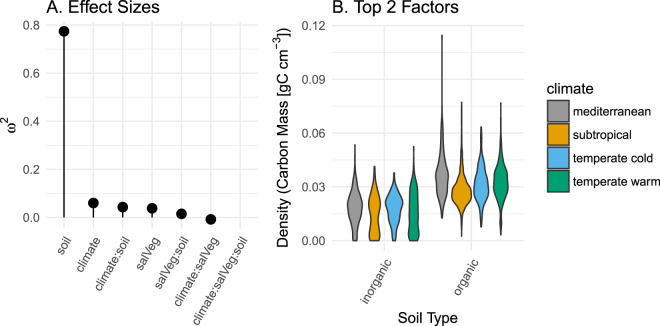
(**A**) Adjusted effect sizes (ω^2^) for each fixed effect in model 1. (**B**) Probability density for carbon stocks across soil type and climate, the two factors with the greatest effect sizes in model 1.

Soil type had the largest effect size in model 1 (ω^2^ = 0.77), with organic soils having higher carbon density than mineral soils (Fig. 5; Supplemental Table 2). Climate type, salinity and vegetation, and interactive effects all had far lower effect sizes (Fig. 5).

An alternative model in which soil type was not considered as an independent variable, which we refer to throughout as ‘model 2’, had far less explanatory power relative to parsimony. Random submitter-effect increased to 13.5 10^−3^ gC cm^−3^ (1.04 σ_c_). Pseudo R^2^ for model 2 decreased from 0.51 to 0.32 and AICc increased from 7278 to 8454. Climate, vegetation and salinity type, and depth interval as well as an interactive effect were present in the most parsimonious version of model 2 (Table 1).

### Non-Spatial Model Validation

**Figure 6:**
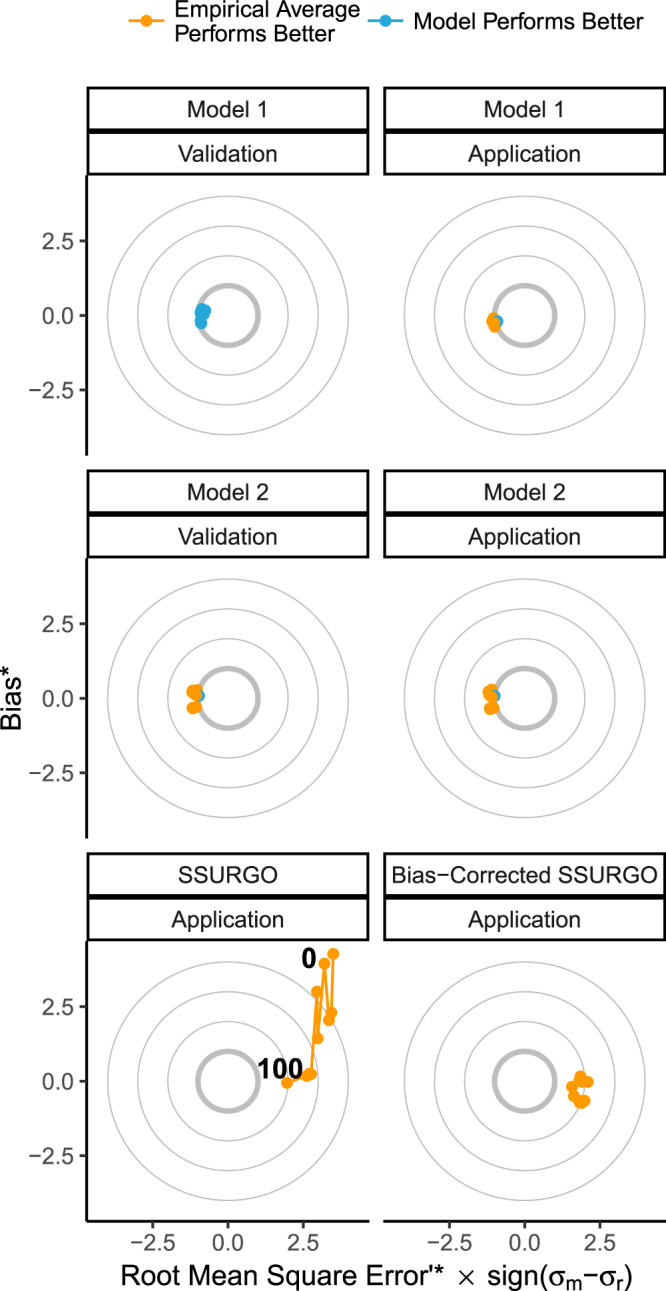
Target Diagrams as outlined by Joliff *et al*.^30^. The x-axis represents unbiased root mean square error (RMSE’), a metric of precision. RMSE’ closer to 0 indicate greater precision. RMSE’ is artificially signed to show whether the modeled (m) or reference (r) dataset has the greater standard deviation (σ). The y-axis shows Bias, a metric of accuracy. Bias values closer to 0 indicate greater accuracy. Positive values indicate the model values are too high, negative values indicate model values are too low. The circles represent total root mean square error (RMSE). All indices have been standardized (*) by σ_r_. RMSE* values less than 1 σ_r_ (bold circle) indicate that the model performs better than the average of the reference dataset. Thinner circles represent RMSE* values of 2, 3, and 4 σ_r_ for reference.

For model 1, which included soil type, modeled values had lower standard deviation than the reference dataset (Fig. 6; Supplemental Table 3). Standardized bias (bias*), a metric of accuracy, and standardized unbiased root mean square error (RMSE*’), a metric of precision, ranged from −0.26 to 0.20 and 0.75 to 0.91 of the reference dataset’s s.d (σ_r_) respectively (Fig. 6). Total normalized RMSE (RMSE*), a metric of total error, was consistently below 1 σ_r_ for all depth intervals (0.77 to 0.92 σ_r_), indicating that the model performed better than the application of the mean from the reference dataset (Fig. 6)^30^.

Similar to model 1 the s.d. of modeled values for model 2, which did not include soil type, were lower than the reference dataset. Bias* ranged from −0.29 to 0.09 σ_r_, however RMSE’* was poorer compared to model 1 ranging from 1.19 to 0.96 σ_r_ (Fig. 6). Model 2 did not consistently perform better than the application of average values, and RMSE* fell below a performance threshold of 1 σ_r_ for only one depth interval (80 to 90 cm; Fig. 6).

### Spatially-Explicit Model Application

While model validation identified potential categorization benefits (e.g. soil type), it did not incorporate any of the uncertainty in the underlying layers needed to create national-scale mapping products. These included SSURGO for soil type (model 1), and the Coastal Change Analysis Program (C-CAP) for salinity and vegetation types (models 1 and 2).

When model 1 was applied using a derivative SSURGO-based organic and mineral-dominated soils map and C-CAP maps for salinity and vegetation classes, precision decreased, and normalized total error increased above the 1 σ_r_ performance threshold (RMSE* = 1.07 to 0.97 σ_r_; Fig. 6; Supplemental Table 3). Bias* remained relatively low but was consistently negative ranging from −0.09 to −0.38 σ_r_. An independent accuracy assessment of our derivative SSURGO-based organic and mineral-dominated soils maps indicated 76.8% total agreement and partially explained the negative bias observed because commission errors and omission errors were asymmetric (Supplemental Table 4). Commission errors were notably high for mineral-dominated soils; 42.8% of the mapped mineral-dominated soils were actually misclassified organic soils. When model 2 was applied, it performed similar to its’ non-spatial validation, with relatively low bias*, but relatively high RMSE*’, and RMSE* that fell below a performance threshold of 1 σ_r_ for only one depth interval (80 to 90 cm).

Applying SSURGO as an independent soils map resulted in mapped values that had a higher s.d. than the reference values (Fig. 6). SSURGO exhibited a depth-wise trend in bias* ranging from 4.27 σ_r_ from 10 to 20 cm and −0.06 σ_r_ at 80 to 90 cm (Fig. 6; Supplemental Table 3). RMSE’* for SSURGO was relatively high compared to the fit models ranging from 3.5 to 1.96 σ_r_ (Fig. 6). RMSE* also exhibited a depth-wise trend, being highest for shallow depths, at 20 to 30 cm exceeding 5.5 σ_r_ (Fig. 6).

We attempted to ‘Bias Correct’ SSURGO bulk density data and found that SSURGO’s organic self-packing density (k1) values range from 0.24 to 0.30 g cm^−3^, with no significant relationship to depth. SSURGO k1 values are greater than twice that of empirical data (k1 = 0.086 to 0.146 g cm^−3^). Bias correcting SSURGO using the known relationship between organic matter content and bulk density^24^ substantially reduced bias, but did not improve precision or reduce RMSE* below the required threshold of 1 σ_r_ (Fig. 6; Supplemental Table 3).

### Ramifications of Model Choice

We mapped 2.67 million hectares (m ha) of coastal wetlands based on national scale maps. Given our most precise, and accurate applied strategy, a simple 27 kg C m^−3^ average carbon mass assumption, we estimate 0.72 (Pg) of C for the top 1 meter of soil. SSURGO soils maps do not perfectly overlap mapped tidal wetlands because of missing or incomplete survey data; overlapping area covered 1.97 m ha (Table 2).

**Table 2:** Total carbon (C) mapped using the four techniques outlined in this paper.

Area (million ha)	CCAP, NWI and SSURGO		CCAP and NWI		Accuracy and Precision Description
	1.97		2.67		
	PgC	Average (kg Cm^−3^)	PgC	Average (kg Cm^−3^)	
Average Carbon Density	0.53	27.0	0.72	27.0	Best performing strategy
Model 1	0.43	22.0	.	.	Accurate but not precise
Model 2	0.37	18.8	0.52	19.4	Accurate but not precise
SSURGO	1.15	58.0	.	.	Positively biased and not precise
Bias-Corrected SSURGO	0.54	27.1	.	.	Accurate but not precise

**Figure 7:**
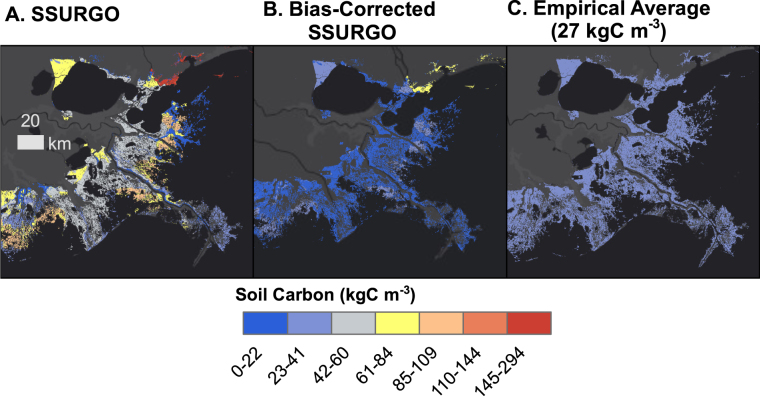
Map showing three alternative mapping techniques for the Louisiana Delta using SSURGO, Bias-Corrected SSURGO, and a null assumption of 27 kg Cm^−3^ overlain on the ESRI © Dark Gray Canvas Basemap. Reprinted with permission from ESRI, ArcGIS, HERE, Garmin, INCREMENT P, © OpenStreetMap contributors, and the GIS user community under a CC-BY license, original copyright 2018.

Comparing all approaches, the SSURGO-limited spatial mapping led to a tidal wetland C stock total of 0.53 Pg C, using the simple mean, and 0.54 using the bias-corrected SSURGO data, whereas models 1 and 2 resulted in slightly lower national-scale carbon stock values (0.43 and 0.37 Pg). In contrast, utilizing unadjusted SSURGO data and maps resulted in a CONUS stock estimate of 1.15 Pg C, thus 54% higher than the approach of applying a single average carbon density. We visualized the comparison between these maps for the Louisiana Delta in Fig. 7.

### Quantitative Data Available to SSURGO

Since we detected negative bias in classifying organic soils using SSURGO organic matter content data and positive bias when calculating carbon density using both SSURGO organic matter content and bulk density data, we reviewed the empirical data that informs SSURGO at the pedon level. The National Cooperative Soil Survey (NCSS)’s pedon database archives quantitative data available to SSURGO soil scientists. One-hundred eleven pedons overlapped mapped tidal wetland area. These included 14 of the 22 tidal CONUS states and were both less numerous and less spatially representative than our empirical dataset. Further, a close inspection of the archived data shows very limited measurements from tidal wetland pedons; approximately one-third of the pedons lack any empirical bulk density or organic carbon data, and most of the remaining pedons are either missing empirical organic carbon or bulk density data, or missing some depth horizon data. Four of the 111 relevant pedons had both organic carbon and bulk density measurements complete and continuous down to 1 m depth (Supplemental Table 5).

## Discussion

In this paper we leverage an extensive dataset (Fig. 1; Supplemental Table 1) to offer feedback on IPCC *Wetlands Supplement* guidance for reporting national soil carbon stocks, and to recommend against the use of categorical mapping strategies in favor of the simplest assumption, assuming an empirical mean carbon density of 27.0 kg C m^−3^ for CONUS tidal wetlands. We also offer insight into potential future research directions for globally-relevant carbon accounting, and encourage of the use of standardized accuracy, precision metrics to intercompare future competing mapping strategies.

### Feedback on IPCC *Wetlands Supplement* Guidance

Our first set of goals were to offer specific feedback on IPCC *Wetlands Supplement* guidance in order to contribute towards future improvements. First, we found that guidance for basic reporting of means and confidence intervals was not appropriate for the distribution of the data. The IPCC *Wetlands Supplement* lists means and confidence intervals for log-transformed data across all carbon stock metrics^10^ (Fig. 2). Log-normal distributions are useful for data that are strictly non-negative and positively skewed. However our soil stock data did not exhibit positive skew and more closely fit a more conventional normal distribution, truncated so that values cannot fall below 0 (Fig. 2). Second, the relationship observed between organic matter and carbon density suggest a lower threshold of >13% organic matter is warranted separating organic soils from mineral soils for tidal wetland soil carbon stocks (Fig. 4). IPCC currently suggests classifying organic soil >20–35% organic matter depending on inundation and clay content. Third, IPCC recommends disaggregating carbon stocks by soil, climate, and salinity and vegetation types^10^. Our analyses show that soil type is by far the most important of these factors, with climate, and salinity and vegetation types, having far less weight (Fig. 5), and not producing predictive models in the absence of reliable soils information (Fig. 6; Table 2).

An important ramification of this synthesis and the organic matter density model is the need for relevant definitions of soil type for carbon stock accounting. We note an empirically-derived 13.2% organic matter threshold may be useful for these explicit carbon accounting purposes and is distinct from the broader taxonomic definition of organic and mineral soils based on geochemical, biogeochemical and hydrologic soil properties. Breithaupt *et al*. similarly recognized the utility of a lower classifying threshold separating mineral, intermediate, and organic soils for C accounting in subtropical mangrove soils^31^.

### The Accuracy and Precision of Soil Carbon Mapping Approaches

Our second goal was to identify optimal strategies for mapping tidal wetland soil carbon stocks that improve accuracy and precision over using a single mean carbon density value. No strategy we applied was both more accurate and more precise than the spatial application of a single mean carbon density value (Fig. 6; Table 2). We suggest that the data distribution reported herein illustrates some fundamentally important accounting principles for tidal wetland soils that merit two separate discussion points. First we find that the mean carbon density is well constrained for tidal wetland soils, both spatially and downcore. Second, we find that for CONUS, proposed categorization and mapping strategies failed to improve precision, and in some cases introduced bias.

#### Mean Carbon Density is Well-Constrained

The average carbon mass (27.0 kg C m^−3^) we found in our large empirical dataset is comparable to multiple previous syntheses in the U.S. and other locations. The mean value we observed using a Tier II (nation-specific) approach is within the confidence intervals for IPCC global default values for salt marshes (25.5 [25.4–29.7 95% CI] kg C m^−3^). Therefore, applying Tier I default using reference carbon stocks provided by the IPCC *Wetland Supplement* would have been reasonable. A recent independent CONUS-wide study supported this estimate reporting an adjusted 28.0 ± 7.8 s.d. kg C m^−2^ to 1 m in soils of saline wetlands^21^. For European tidal marshes, van Broek *et al*.^32^ report a mean of 26.1 kg C m^−3^. In Southeastern Australia mangroves and marshes have a mean carbon density of 25.3 kg C m^−3 ^^33^, with no effect of vegetation type but geomorphic influences within drainages.

The convergence of carbon density across sites both simplifies accounting approaches and confirms a narrow set of conditions that constrain the relationship of organic matter to bulk density in tidal wetland soils. We hypothesize that decomposition allows compaction within these sites; lower organic matter values down core are on average offset by increasing bulk density (Fig. 2). The lack of a depth effect in model 1 further illustrates this potential feedback and calls into question the spatial variability and downcore trends apparent in the SSURGO tidal wetland dataset (Fig. 6).

The concepts introduced by the ideal mixing model and the organic matter density model provide some context for the low variability in observed carbon density. Macreadie *et al*.^34^ report a wide range of soil carbon densities (1.4–96.2 kg C m^−3^) across 323 samples from Australian salt marshes, but the distribution is strongly weighted toward mineral soils, less than 14% organic matter. The majority of the variability occurs within these mineral soils, and appears to be related to grain size associated with geomorphic conditions^33^. Similar to our study, a narrow distribution is observed in Australian salt marshes, around a relatively low mean of 16.5 ± 0.7 s.e. kg C m^−2 ^^34^. This is consistent with the assumption of the organic matter density model, that carbon density should vary over a fairly narrow range, predictably increasing only along a spectrum of more to less mineral-dominated soils between 0 and 13.2% organic matter (Fig. 4). Above this threshold, organic matter approaches its maximal self-packing density in tidal wetlands (k1 = 0.098 g cm^−3^), and carbon mass changes less with the organic/inorganic composition ratio. This established an effective upper limit of 0.048 gC cm^−3^ (48.0 kg m^−3^) that highly organic soils should not theoretically exceed on average. We note that while the assumption of an additive relationship between organic and inorganic fractions was useful for describing soil properties in this synthesis, there is an argument and some evidence that this assumption may not apply to karstic mangrove soils of the Everglades and Gulf of Mexico^31^.

#### Currently Available Soil Carbon Mapping Strategies Lack Necessary Precision

Our analysis of competing mapping and modeling strategies indicated that many geographic categories previously emphasized in the literature (such as vegetation, climate and depth) may be less important than previously assumed (Fig. 5). Relying on positively biased soils maps could positively bias future emissions accounting if they were used for that purpose (Fig. 7; Table 2).

Chmura *et al*.^1^, reported higher carbon densities for mangrove soil globally, and the IPCC *Wetlands Supplement* recommends a higher default estimate for mangroves compared to tidal marshes. However, after controlling for random submitter effect and soil type, we observed little effect of vegetation type on carbon density (Fig. 5). These results together suggest that mapping the conditions that promote organic or mineral-dominated soil formation independent of dominant vegetation type should be an important future priority for mapping tidal carbon stocks.

Hinson *et al*.’s^23^ SSURGO-based approach exhibits a depth-specific trend and elevated carbon densities compared to our empirical and model approaches (Fig. 6). We estimate that their average carbon stocks are over-reported at 170% for the top 15 cm, 110% for the top 30 cm, and 50% for the top 1 m. These densities and depth trends are not observed in our empirical data nor in Nahlik and Fennessy’s^21^ independent analysis using soil data of the EPA National Wetland Condition Assessment (n = 319 cores to 120 cm). These apparent biases may be a result of low data availability to SSURGO soil scientists (Supplemental Table 5) who have traditionally focused on mapping CONUS soils for agricultural and and development purposes^27^. The NCSS Soil Survey Handbook refers to organic tidal wetland soils as lacking specific features and being difficult to survey^35^.

Soil type was the most important category tested, having far more explanatory power in model 1 than any other environmental metric (salinity, vegetation, or climate type). Although standardized precision, accuracy, and total error metrics indicated that model 1, dominated by soil type effects, had the potential to outperform the use of a single average carbon density value, in practice it did not. Our accuracy assessment indicated that the CONUS soil map misclassified as many as 42.8% of organic soil observations as mineral (Supplemental Table 4), and thus introduced a negative bias. Our data implies that improving the accuracy of the underlying organic and mineral soils maps would have the greatest overall effect on reducing carbon stock mapping errors.

To illustrate the implications of over-estimated soil stocks, we mapped the stocks associated with 3 different approaches in the dynamic, loss-prone wetlands of Louisiana (Fig. 7). For the Barataria basin alone, shown in Fig. 7, SSURGO-based results from suggest a far higher and more variable soil stock (total = 0.0575 PgC; range = 3–92 kg Cm^−3^) than that estimated from either bias-corrected SSURGO (total = 0.0158 PgC; range = 3–37 PgC) or from our simple empirical mean approach (0.0283 PgC). Barataria Basin has been documented to have had some of the highest wetland loss rates in Louisiana, (−12.10 ± 2.51 km^2^ yr^−1^ from 1985–2010)^36^. Assuming emissions commensurate with that loss rate using watershed averages as in Hinson *et al*.^23^ would have resulted in estimated emissions of 18.3 m tonnes CO_2_ emitted according to the null map, 10.2 for Bias-Corrected SSURGO and 37.1 for SSURGO from 1985 to 2010. Our previous analyses showed that on the national scale SSURGO and bias-corrected SSURGO lack precision compared to the null strategy (Fig. 6; Table 2). However, this exercise shows that lack of accuracy and precision could also potentially lead to under or overestimation of stocks or emissions at a local scale.

### Implications for Future Research

Some suggestions we have for future studies include closer attention paid to methods and lab-specific error, better mapping of the depths of stocks affected by degradation events, and more explicitly connecting variation in soil carbon to ecologically and biogeochemically relevant processes.

For accuracy assessment purposes we made the assumption that reference values were an improvement over mapped values, and that they approximated ‘true values’^37^. However, random submitter specific error for model 1 (0.48 σ_c_ or 6.2 kg Cm^−3^) was not unsubstantial. Controlled comparative lab studies show that inter-lab variance in loss-on-ignition is higher than intra-lab differences, and lab-specific bias is associated with sample size, ignition time, and ignition temperature^38^. Future studies could attempt to further control for these three variables.

Rather than focusing only on the top 1 m of soil using the IPCC default assumption for anthropogenic activity^10^, future studies could improve total soil stock estimates by more explicitly measuring and reporting deposit depth. In our empirical dataset most cores were from shallow coring efforts (e.g. 24 cm for the Louisiana Coastwide Reference Monitoring System [CRMS]). Comparatively few studies reported reaching bedrock or non-marsh sediment interface (Supplemental Table 1). Of the studies that did report these measures maximum depth ranged from 1550 cm for a site on the Nanticoke River in the Chesapeake Bay^39^ to 23 cm for a thin mangrove peat in the Florida Everglades^31^. We recognize explicitly that the depths of deposits relevant for quantifying total tidal wetland soil carbon stock in CONUS are a large unknown, and that our estimates likely underestimate the deep deposits in currently stable tidal wetlands. Likewise, shallow peat profiles from CONUS mangroves may be a locally specific phenomenon, and should not be generally assumed as tropical mangrove peats can reach great depths, for example 600 cm in the Florida Keys and up to >1200 cm in Belize^40^.

Future studies could focus on identifying spatial scales that are relevant to the processes of organic matter accumulation and loss in tidal wetland soils. Broad biogeographic drivers are often emphasized in the literature, for example, carbon stock differences between arid and humid climates^41,42^. Chmura *et al*.^1^, for example, reported negative correlations between carbon density and latitude, which could imply some relationship between soil carbon burial properties and growing season photosynthesis observed in marsh grasses^43^ and peat mosses^44^. However, along the U.S. Atlantic Coast, the geographic source of much of the synthetic data, latitude is also intercorrelated with tidal amplitude and relative sea-level rise, variables which strongly affect wetland soil formation^12,24^. Geomorphic drivers are more difficult to map than climate variables but may exert an outsized influence on soil organic matter.

In addition to providing empirical and theoretical guidance for improved carbon accounting, this paper demonstrates a new application of existing statistical metrics and a visualization technique^30^. Although these techniques of assessing ‘model skill’ have been applied to simulations models in coastal carbon shelf science^45^, as far as we are aware, they have not yet been widely adopted in coastal wetland carbon stocks mapping. We encourage the use of these standardized metrics in future intercomparisons as science and mapping of tidal soil carbon stock improve.

## Conclusion

Given a single average carbon stock estimate, an assumption of 1 m depth and an area of 2.67 m ha, we estimate CONUS tidal wetlands contain 0.72 Pg of soil organic carbon. Soil carbon stocks, based on a large empirical dataset, were far lower on average and varied far less spatially and with depth than stocks calculated from available national-scale soils maps. Soil type was the single most important driver of variability, with little evidence of climate, vegetation, or salinity type influencing C stocks enough to produce a predictive model. For tidal wetlands soils with >13.2% organic matter, organic matter density increases very little as organic fraction increases. We quantified upper limits on mean tidal soil carbon stock defined by organic matter’s self-packing density. Although tidal wetland carbon stocks are greater in organic compared to mineral soils^10^, we cannot readily apply that knowledge given the limited accuracy and precision of available soils maps. Relying on a single average value (27 kg C m^−2^) was the most precise approach we tested, and importantly, is unlikely to substantially bias coastal carbon monitoring efforts. Overall, our analysis supports the use of a simpler metric for both stock assessment and estimating emissions. However, we also present metrics by which future efforts could be assessed and intercompared for predicting carbon distributions at system-relevant spatial scales.

## Methods

### Soil Core Dataset

We collected disaggregated depth profile data for 1959 cores, with associated metadata. Cores were collected from the literature^26,39,46–69^, reports^70–72^, public databases^73–75^, unpublished data in preparation for peer review submitted by co-authors or generous members of the scientific community (Supplemental Table 1). CRMS cores were included if they intersected tidal wetlands as mapped by the National Wetlands Inventory (NWI). Cores ranged from 5 to 1550 cm in length, with a mean length of 55 cm. The median of the length dataset was 24 cm because a great deal of the dataset was made up of the Louisiana CRMS dataset (Supplemental Table 1).

Bulk density for all cores was measured gravimetrically. We assumed core compaction was minimal, although different authors dealt with this assumption in various ways. Drying temperature and time varied by lab and submitter, ranging from 60 to 105 °C. Drying times ranged from 96 to 24 hours or otherwise specified ‘overnight’ or ‘to a constant volume’. Many cores had % organic matter data measured as the fraction dry mass determined by loss-on-ignition (LOI)^38^. Ignition-temperature and time varied based on submitter, ranging from 400 to 550 °C, and 1 to 16 hours.

Six hundred two cores reported measured organic carbon (%), 475 cores in addition to organic matter and 127 without. Carbonates were often removed physically by applying dripped dilute acid or fumigating with concentrated acid (n = 138). Four hundred twenty five cores reported organic carbon without specifying acid treatments or reporting other strategies for carbonate removal, which is typically most important in karst embayments. Thirty nine of the cores in the dataset measured % total carbon rather than organic carbon, but we assumed that carbonate was a minimal contribution to the total.

### Empirical OC Conversion Factor

We independently verified a function for predicting organic carbon from organic matter published by Craft *et al*.^76^. We used a subset of paired data points from six data sources that had published organic matter content by LOI and organic carbon by elemental analysis^26,47,55,70,71,77^. Studies spanned CONUS including the Pacific Northwest, San Francisco Bay, Louisiana, the Everglades, and Long Island Sound. We modeled organic carbon as a quadratic function of organic matter using multi-model inference, algorithm which selects optimal models based on Akaike’s Information Criteria for small datasets (AICc)^78,79^ in ‘R’^80^. A quadratic function outperformed a linear function in terms of parsimony relative to explanatory power (Eq. 1; Supplemental Fig. 2, R^2^ = 0.932, n = 1594).

1 $$OC=0.074\pm 0.014\,O{M}^{2}+0.0421\pm 0.012\,OM-0.0080\pm 0.0021$$

In which:

OC = fraction organic carbon.

OM = fraction organic matter.

### Empirical Dataset Calculations

We summarized empirical data bulk density, organic matter, and organic carbon content across 10 cm increments down to 1 m using a depth weighted average, normalizing sampling interval to 1 cm increments and summing across the 10 cm depth intervals. If the deepest sample depth covered >50% of the depth increment the carbon mass, bulk density and organic matter were extrapolated to the bottom of the interval. If not the increment was considered a ‘no data’ value. If a cores had LOI data we estimated organic carbon from Eq. 1 even if organic carbon was additionally measured or estimated for the study. We estimated carbon density from LOI and organic carbon only if there were no LOI data present.

We described central tendency and spread by fitting probability densities to histograms for both a normal distribution and a log-normal distribution. For the log-normal distribution we assumed all zero values were not truly zero but below a detection limit of 0.1 kg C m^−3^, so we recast 0 values as 0.01 kg C m^−3^ for to generate the log-normal distribution. This was for the sake of the exercise in visually comparing the two datasets, and zero values were not recast for reporting the mean and s.d. assuming the normal distribution.

We randomly sorted cores into two independent subsets, a calibration dataset used for generating averages uncertainties and fitting models, and a reference dataset used for performing accuracy assessments and calculating model performance statistics.

### The Ideal Mixing Model and Organic Matter Density Model

We used a variant of the ideal mixing model applied by Morris *et al*.^24^, herein referred to as the organic matter density model, to describe how organic matter density varies along a spectrum of soil types from purely mineral to purely organic (Fig. 4). The ideal mixing model has an advantage over linear correlations^25,26^ because it physically describes the additive and volume-limited nature of the organic and mineral fractions that form a soil matrix^24^. Bulk density is a function of fraction organic matter as well as the organic (k1) and mineral (k2) self-packing densities, conceptually the average density of pure organic and mineral matter respectively (Eq. 2).

2 $$BD=\frac{1}{\frac{OM}{k1}+\frac{(1-OM)}{k2}}$$

In which:

BD = bulk density.

OM = the organic matter fraction.

k1 and k2 refer to the self-packing density of pure organic matter and pure mineral matter respectively (g cm^−3^).

Organic self-packing density (k1) has important practical and theoretical implications for tidal wetland carbon accounting because it defines a hypothetical upper limit of carbon mass in organic soils when OM = 1. OM density should approach, but on average, not exceed, organic self-packing density (k1) in organic soils. We refer to this equation throughout as the Organic Matter Density model (Eq. 3).

3 $$OMD=\frac{OM}{\frac{OM}{k1}+\frac{(1-OM)}{k2}}$$

In which: OMD = Organic matter density.

For the calibration dataset, we fit the ideal mixing model to generate p-value^81^. We refined model parameters k1 and k2 and generated uncertainty estimates using a bootstrapped approach including 1000 iterations^81^.

### Mixed Effects Models and Power Analysis

We defined organic and mineral soils using a bootstrapped piecewise linear regression^82^ for all cores and depth classes together.

We mapped climate zone using the same standards as the EPA’s Greenhouse Gas Inventory^83^. We used four classes: mediterranean, subtropical, temperate cool, and temperate warm. Mediterranean was defined as within the state of California and south of 40° latitude. Subtropical was defined as the Gulf Coast and the Atlantic coast of Florida south of 30° latitude. Temperate warm included the Atlantic coast between 30 and 40° latitude. Temperate cool included the Pacific and Atlantic Coasts north of 40° latitude. We realize that these are do not perfectly match Köppen-Giel climate zones^84^, but we applied them to remain consistent with other community efforts^83^.

We also tested combined salinity and vegetation type as potentially predictive (estuarine emergent, estuarine forested/scrub shrub, palustrine emergent, and palustrine scrub/shrub). Vegetation categorization differs slightly from that recommended by the IPCC (marsh and mangrove). We did this to match classifications in the Coastal Change Analysis Program (C-CAP) a Landsat-based land cover and change product. Because C-CAP defines forested and scrub/shrub based on shrub or tree heights^85^, and that data was not available for our soil core database, we combined forested and scrub/shrub categories.

Because this effort synthesized data from multiple sources, and measurements such as LOI and dry bulk density can have laboratory specific biases, we integrated a random ‘submitter’ effect into our modeling structure. We considered a submitter to be the first author of an associated peer-reviewed publication, or the individual or organization responsible for actively managing the original dataset. We generated submitter codes as the last name of the submitter or commonly used acronym for an organization. Random effects were combined with fixed effects and all potential interactions using the R packages ‘lme4’^86^, using the syntax in Eq. 4.

4 $${carbonDensity} \sim {lmer}({climate}\times {depth}\times {salVeg}\times {soil}+({1}|{submitter}))$$

In which:

lmer is a linear mixed effects model.

climate is one of four mapped climate zones

salVeg is one of four mapped salinity and vegetation types

depth is one of the 10 cm depth increments down between 0 and 1 m

soil is either organic or inorganic dominated

The intercept of the linear model is conditional on random variation associated with the data submitter

We used multi-model inference^78,79^ in R to test model fit relative to parsimony as measured by AICc, for all possible permutations of factors. Specifically, we used the ‘dredge’ function in the ‘MuMIn’ package^79^. We selected the model with the highest ranking Akaike weight as ‘model 1’. We calculated the pseudo R^2^ value for model 1 also using the R package ‘MuMIn’^79^.

We did two things to quantify and contextualize effect sizes for the various fixed effects. First we calculated an adjusted effect size (ω^2^) using the ‘anova_stats’ function in the R package ‘sjstats’^87^. We also repeated the dredge process described previously on a version of equation 4 that did not include soil type as a variable, model 2.

### Areas of Interest

We calculated wetland area using both 2006–2010 C-CAP^85^ raster maps as well as the NWI^88^ vector data. We downloaded CCAP 2006–2010 change data for all 22 coastal CONUS U.S. states. We extracted all pixels that were coded as estuarine emergent, scrub/shrub, or forested in 2006 or 2010. For any remaining pixels coded as palustrine emergent, forested, or scrub/shrub in 2006 or 2010 we included pixels if they fell within the boundaries of mapped tidal wetlands according to NWI polygons coded with tidal hydrology modifiers. According to the National Resources Conservation Service (NRCS) soil survey boundaries, there are 288 survey zones overlapping mapped tidal wetlands^89^. Of these 288 zones, there were sixteen zones that had incomplete or missing data.

### Weighted Averages for SSURGO Hydric Soil Components

SSURGO ‘map units’ represent the spatial extents of soils using mapping techniques, soil surveys, and expert judgment taking into account landscape factors. SSURGO contains multiple linked data tables associated with those map units. Each map unit may have one or more components, soil descriptions that make up a percent of that map unit, indicated by the ‘component percent’. Each component can have one or more ‘horizons’, depth intervals, which contain organic matter content and bulk density data^27^.

We downloaded all SSURGO maps and tables corresponding to soil survey areas intersecting mapped tidal wetlands^89^. We extracted all SSURGO map units intersecting mapped tidal wetlands from NWI, and further extracted all components categorized as ‘hydric’. If bulk density data was present, but organic matter was not, organic matter was assumed to be 0%. If both values or bulk density were missing, the horizon was interpreted as a ‘no data’ value.

To summarize data at the map unit scale we first calculated depth weighted averages for organic matter content, bulk density, and organic matter density separately based on each component’s separate horizon data. We then summarize each of these variables for map units as the weighted average of all the components based on their reported component percent^28^. We did not perform rock fragment corrections, as it is not applicable to tidal wetland soils^29^. We estimated carbon density using the van Bemmelen factor (0.58 gOC gOM^−1^), as that is the recommended conversion factor for SSURGO^27,28^. Carbon density (gC cm^−3^) was converted to mass area^−1^ by multiplying by the depth interval (10 cm) and converting to kg C m^−2^ (1 kg 1000 g^−1^ and 10^3^ cm^2^ m^−2^). We also assigned SSURGO map units a binary classification of ‘mineral-’ or ‘organic-dominated’ based on a detected empirical threshold of 13.2% organic matter.

### Model Validation, Model Application, and SSURGO Application

Reference dataset members were additionally screened so that low-quality latitude-longitude coordinates were excluded (n = 960 cores). Location information was coded as coming from GPS measurements, map figures or site descriptions. If positional information was not able to be effectively matched to a SSURGO map unit they were excluded.

We assessed ‘model skill’ at two phases, what we refer to throughout as a validation phase, and an application phase. For model validation we modeled carbon density based on ‘true values’. We used the ‘R’ predict function using only the fixed effects from the mixed effects models 1 and 2. This allowed us to determine whether or not the models were overfit or unduly influenced by outliers in the calibration dataset. For model application we modeled carbon density based on ‘mapped’ values following the same procedure for model validation, except using mapped values. Application compounded uncertainty in both the models and the underlying data products used to apply the model.

To run models that took soil type, climate, vegetation and salinity, and/or depth interval as inputs, each reference dataset core was assigned ‘true values’ based on field descriptions and empirical data, and a ‘mapped values’ based on SSURGO for soils and C-CAP for salinity and vegetation. All spatial statistics were done in ArcGIS Pro^90^. We assumed no mapping errors in climate zone or depth intervals.

We evaluated models using accuracy and precision metrics, normalized bias (bias*) and unbiased root mean square error (RMSE*’) (Eq. 5–8)^30^. We also calculated total normalized root mean square error (Eq. 9). Because RMSE* is normalized to the s.d. of the reference dataset, values that are less than 1 indicate that the model performs better than the average^30^. Values greater than one indicate the average performs better.

5 $${B}^{\ast }=\frac{{\mu }_{m}-{\mu }_{r}}{{\sigma }_{r}}$$

In which: B* = normalized bias.

μ_m_ and μ_r_ = means of the modeled and reference values respectively.

σ_r_ = standard deviation of the reference values.

6 $$R=\frac{\frac{1}{n}\,{\sum }_{i=1}^{n}({m}_{i}-{\mu }_{m})({r}_{i}-{\mu }_{r})}{{\sigma }_{m}{\sigma }_{r}}$$

In which:

R = correlation coefficient.

n = number of data points.

m_i_ = ith modeled value.

r_i_ = ith reference value.

σ_m_ = s.d. of modeled values.

7 $${\sigma }^{\ast }=\frac{{\sigma }_{m}}{{\sigma }_{r}}$$

In which: σ* = normalized s.d.

8 $$RMS{E}^{\ast ^{\prime} }=\sqrt{1+{\sigma }^{\ast 2}+2{\sigma }^{\ast }R}$$

In which: RMSE*’ = standardized unbiased root mean square error.

9 $$RMS{E}^{\ast }=\sqrt{RMS{E}^{\ast ^{\prime} 2}+{B}^{\ast 2}}$$

In which: RMSE* = normalized total root mean square error.

In addition to model 1 and 2 we performed these validation metrics of bias*, RMSE*’ and RMSE* on two different applications of SSURGO. First, we validated SSURGO as described above. However we detected a positive bias. Second, we attempted to ‘bias correct’ SSURGO bulk density using the ideal mixing model (Eq. 2) fit to both the calibration dataset and SSURGO. We fit the ideal mixing model to SSURGO OM and BD and calculated the residuals [Eq. 8] and standardized residuals (Eqs 10, 11) of all points. We fit the ideal mixing model to the calibration data and calculated an empirical k1, k2, and the standard deviation of residuals. We then recalculated a ‘corrected’ BD from the modeled BD based on empirical k1 and k2, with new residuals calculated from the standardized SSURGO residual and s.d. of residuals from the empirical data (Eq. 12). Therefore the mean and variance of empirical data are mimicked by SSURGO, with the relative pattern of SSURGO anomalies still in place. Any negative BD values resulting from this shift were assigned the minimum SSURGO BD data value before the bias correction process.

10 $${r}_{i}=B{D}_{i}-\frac{1}{\frac{O{M}_{i}}{k{1}_{s}}+\frac{(1-O{M}_{i})}{k{2}_{s}}}$$

In which:

r_i_ = residual of data point i.

BD_i_ = The i th bulk density.

OM_i_ = The i th organic matter fraction value.

k1_s_ and k2_s_ = the organic and inorganic self-packing densities fit to SSURGO data.

11 $${r}_{i}^{\ast }=\frac{{r}_{i}}{{\sigma }_{s}}$$

In which:

r_i_* = standardized residual of data point i.

σ_s_ = the standard deviation of residuals for SSURGO.

12 $$CB{D}_{i}=\frac{1}{\frac{O{M}_{i}}{k{1}_{c}}+\frac{(1-O{M}_{i})}{k{2}_{c}}}+{r}_{i}^{\ast }{\sigma }_{c}$$

In which:

CBD_i_ is the ‘corrected’ bulk density value for point i.

k1_c_ and k2_c_ = the organic and inorganic self-packing densities fit to calibration data.

σ_c_ is the standard deviation of residuals for calibration dataset.

In the process of evaluating SSURGO we observed and hypothesize that there was additional bias in the classification of organic and mineral soils. To determine whether or not SSURGO data can be useful for generating maps compared to applying carbon mass defaults from our calibration dataset, we classified all SSURGO and validation depth intervals as organic or mineral soils according to IPCC definitions. We calculated accuracy assessment statistics including ‘user’s accuracy’, ‘producer’s accuracy’, and ‘total accuracy’^37^ (Supplemental Table 4).

We classified maps qualitatively using Bias* and RMSE*’ as recommended by IPCC^20^. If bias* were consistently less than 1 σ_r_ for all depth increments, we classified the model as accurate. If RMSE*’ were consistently less than 1 σ_r_ we considered the map to be precise (Table 2).

### Data Coverage in the NCSS Pedon Database for Tidal Wetlands

While assembling SSURGO data to test the efficacy of using it to generate improved carbon stock estimates, we made extensive observations and independently vetted the the empirical datasets available to soil scientists who populate SSURGO with values. The National Cooperative Soil Survey (NCSS) Pedon Database is a resource available to soil scientists; reports contain field and lab descriptions of pedons, three-dimensional soil structures that make up the most basic and disaggregated unit of soil abstraction in the USDA spatial data product hierarchy^91^. The database contains 111 pedons that overlap tidal wetlands as mapped by NWI (as of April 2017).

### Data availability Statement

Detailed summary statistics and figures are available in the supplemental information. Soil carbon maps based on the Soil Survey Geographic Database, salinity and vegetation maps, and modeling efforts are available via the Oak Ridge National Laboratory’s Distributed Active Archive Center (DAAC) for biogeochemical dynamics (10.3334/ORNLDAAC/1612). Soil core data that is from previously published information will be made immediately available via the Coastal Carbon Research Coordination Network (10.25572/ccrcn/10088/35684). Soil core data from previously unpublished sources that are currently ‘in-preparation’ or ‘in-review’ will be made public via the Coastal Carbon Research Coordination network pending their status change to ‘in-press’. Until the time that the full dataset is made public, previously unpublished soil core data inquiries will be referred to the original data submitters as listed in Supplemental Table 1.

## Electronic supplementary material

Supplemental Methods

Supplementary Dataset 1

Supplementary Dataset 2

Supplementary Dataset 3

Supplementary Dataset 4

Supplementary Dataset 5
