# Author Correction: Accuracy and Precision of Tidal Wetland Soil Carbon Mapping in the Conterminous United States

**DOI:** 10.1038/s41598-018-33283-4

**Published:** 2018-10-09

**Authors:** James R. Holmquist, Lisamarie Windham-Myers, Norman Bliss, Stephen Crooks, James T. Morris, J. Patrick Megonigal, Tiffany Troxler, Donald Weller, John Callaway, Judith Drexler, Matthew C. Ferner, Meagan E. Gonneea, Kevin D. Kroeger, Lisa Schile-Beers, Isa Woo, Kevin Buffington, Joshua Breithaupt, Brandon M. Boyd, Lauren N. Brown, Nicole Dix, Lyndie Hice, Benjamin P. Horton, Glen M. MacDonald, Ryan P. Moyer, William Reay, Timothy Shaw, Erik Smith, Joseph M. Smoak, Christopher Sommerfield, Karen Thorne, David Velinsky, Elizabeth Watson, Kristin Wilson Grimes, Mark Woodrey

**Affiliations:** 1 Smithsonian Environmental Research Center, Edgewater, USA; 2 USGS, National Research Program, Water Resources Division, Menlo Park, USA; 3 USGS, Volunteer, Sioux Falls, USA; 4 Silvestrum Climate Associates, LLC, Mill Valley, USA; 5 University of South Carolina, Columbia, USA; 6 Florida International University, Miami, USA; 7 University of San Francisco, San Francisco, USA; 8 USGS, California Water Science Center, Sacramento, USA; 9 San Francisco State University and San Francisco Bay National Estuarine Research Reserve, San Francisco, USA; 10 USGS, Woods Hole Coastal and Marine Science Center, Woods Hole, USA; 11 USGS, Western Ecological Research Center, Vallejo, USA; 12 University of South Florida, Tampa, USA; 13 Coastal and Hydraulics Laboratory, U.S. Army Engineer Research and Development Center, Vicksburg, USA; 14 University of California, Los Angeles, USA; 15 Guana Tolomato Matanzas National Estuarine Research Reserve, Ponte Vedra Beach, USA; 16 Delaware National Estuarine Research Reserve, Dover, USA; 17 Asian School of the Environment, Nanyang Technical University, Singapore, Singapore; 18 Earth Observatory of Singapore and Nanyang University, Singapore, Singapore; 19 Florida Fish & Wildlife Conservation Commission, Fish & Wildlife Research Institute, St., Petersburg, USA; 20 Virginia Institute for Marine Sciences, Gloucester Point, USA; 21 North Inlet-Winyah Bay National Estuarine Research Reserve, Georgetown, USA; 22 University of Delaware, School of Marine Science and Policy, Newark, USA; 23 Department of Biodiversity, Earth & Environmental Sciences and The Academy of Natural Sciences, Drexel University, Philadelphia, USA; 24 University of the Virgin Islands and Wells National Estuarine Research Reserve, St. Thomas, USA; 25 Grand Bay National Estuarine Research Reserve and Mississippi State University, Moss Point, USA

Correction to: *Scientific Reports* 10.1038/s41598-018-26948-7, published online 21 June 2018

This Article contains an error in Equation 1.

$$OC=0.074\pm 0.014O{M}^{2}+0.0421\pm 0.012OM-0.0080\pm 0.0021$$

should read:

$$OC=0.074\pm 0.014O{M}^{2}+0.421\pm 0.012OM-0.0080\pm 0.0021$$

