# Supplementary material for: Accuracy and Precision of Tidal Wetland Soil Carbon Mapping in the Conterminous United States
Source: Sci Rep. 2018 Jun 21;8:9478. doi: 10.1038/s41598-018-26948-7 (PMC6013439; doi:10.1038/s41598-018-26948-7)
Supplement: Supplementary file 1 — Supplemental Methods [file 41598_2018_26948_MOESM1_ESM.pdf]

Accuracy and Precision of Tidal Wetland Soil Carbon Mapping in the Conterminous United States

James R Holmquist *[1], Lisamarie Windham-Myers [2], Norman Bliss [3.], Stephen Crooks [4], James Morris [5], J Patrick Megonigal [1], Tiffany Troxler [6], Donald Weller [1], John Callaway [7], Judith Drexler [8], Matthew C Ferner [9], Meagan E Gonneea [10], Kevin D Kroeger [10], Lisa Schile-Beers [1], Isa Woo [11], Kevin Buffington [11], Joshua Breithaupt [12], Brandon M Boyd [13], Lauren N Brown [14], Nicole Dix [15], Lyndie Hice [16], Benjamin P Horton [17, 18], Glen M MacDonald [14], Ryan P Moyer [19], William Reay [20], Timothy Shaw [17], Erik Smith [21], Joseph M Smoak [12], Christopher Sommerfield [22], Karen Thorne [11], David Velinsky [23], Elizabeth Watson [23], Kristin Wilson Grimes [24], Mark Woodrey [25]

* Corresponding Author: HolmquistJ@si.edu
1. Smithsonian Environmental Research Center
2. USGS, National Research Program, Water Resources Division
3. USGS, Volunteer
4. Silvestrum Climate Associates, LLC
5. University of South Carolina
6. Florida International University
7. University of San Francisco
8. USGS, California Water Science Center
9. San Francisco State University and San Francisco Bay National Estuarine Research Reserve
10. USGS, Woods Hole Coastal and Marine Science Center
11. USGS, Western Ecological Research Center
12. University of South Florida
13. Coastal and Hydraulics Laboratory, U.S. Army Engineer Research and Development Center
14. University of California, Los Angeles
15. Guana Tolomato Matanzas National Estuarine Research Reserve
16. Delaware National Estuarine Research Reserve
17. Asian School of the Environment, Nanyang Technical University, Singapore
18. Earth Observatory of Singapore and Nanyang University
19. Florida Fish & Wildlife Conservation Commission, Fish & Wildlife Research Institute
20. Virginia Institute for Marine Sciences
21. North Inlet-Winyah Bay National Estuarine Research Reserve
22. University of Delaware, School of Marine Science and Policy
23. Department of Biodiversity, Earth & Environmental Sciences and The Academy of Natural Sciences, Drexel University
24. University of the Virgin Islands and Wells National Estuarine Research Reserve
25. Grand Bay National Estuarine Research Reserve and Mississippi State University

1. Supplemental Information

1.1. Generating Final Mapped Products

For SSURGO organic and inorganic soils extents at each 10 cm increment, and SSURGO and bias-corrected SSURGO carbon stocks down to 1 m, we joined a geodatabase file containing spatial information on soil map unit extent with tables containing 1 m depth totals, indexed by the map unit code. We rasterized polygons and clipped resulting rasters using the mapped extent of tidal wetlands and SSURGO. All layers had 30 m resolution, the same coordinate system, and ‘snapped to’ pixel extent, matching C-CAP.

We created final map products for the top meter of soil for the null model (average 27

kgC m^{-3}), and model 1 and 2. For the null model and model 2 we made two versions, one clipped to the intersection of C-CAP wetlands and NWI tidal surfaces, and a second clipped to the intersection of C-CAP wetlands, NWI tidal surfaces, and SSURGO map units with hydric soils. For model 1 and the two applications of SSURGO, maps were created using the latter extent.

Since model 1 was depth independent and soils data were required, we created a intermediate raster data layer representing the number of organic and inorganic depth horizons. We created an index layer so that each combination of salinity, vegetation, and climate zone had a unique integer. We then generated a table of associated values for each of model 1's potential outputs to join to the index layer. We did this for organic and inorganic soils separately, multiplied by the number of appropriate soil depth horizons and summarized the two layers to calculate the total carbon mass down to 1 m.

Since model 2 was depth dependent and soil type independent. We joined the unique climate, salinity and vegetation index layer to a table of the predictions for each class, with all depth interval predictions, summed to one meter.

1.2. List and Description of Supplemental Files

Supplemental Table 1: Summary information for all data assembled from literature review, and coauthor and community submissions

Supplemental Table 2: ANOVA results from the fixed effects predicting carbon mass as a function of soil type, depth, vegetation and salinity subtypes, with all interactive effects possible for models 1 and 2

Supplemental Table 3: Performance statistics from comparisons between fit model and SSURGO-based maps and the reference dataset

Supplemental Table 4: Accuracy Assessment Summary for SSURGO maps categorized based on classifying organic soils as >13.2% OM. This assessment includes all validation data intersecting SSURGO hydric map units and all complete 10 cm depth intervals down to 1 m.

Supplemental Table 5: List and summaries of the NCSS pedon reports from pedons intersecting mapped tidal wetlands
